# Treatment of Diptheria

**Published:** 1871-03

**Authors:** J. T. Costen

**Affiliations:** Newton, Md.


### TREATMENT OF DIPHTHERIA.

BY J. T. COSTEN, M. D, OF NEWTON, MD.

During the past year we have had prevailing in our town, and over a great portion of the county, an epidemic of diphtheria of a malignant character.

The treatment given in books on that subject appeared to have no control over it whatever. Indeed I think the medicine that is most popular, muriated tincture of iron, has a bad effect; all of those who could express their feelings, said they felt worse after taking it.

After spending some time trying such medicines as had been recommended by others, and finding their effect was not that desired, and seeing that the membrane was the cause of death in every case, I resolved to find, if possible, a medical agent that would destroy it, believing that it would also neutralize it in its circulation, and thereby check its formation. I took some membrane from the throats of my patients, and subjected it to the action of all the medicines that I had reason to believe would affect it. I tried the acids, which only made it harder ; notwithstanding I have seen it stated that lactic acid would dissolve it. I then tried the alkalies and other medicines. Bicarbonate of soda and liquor potassæ being the only ones that had the desired effects. Bicarbonate of soda in concentrated solution dissolves it slowly, liquor potassæ as soon as it comes in contact.

I at first gave the bicarbonate of soda in very large doses as an internal remedy, at the same time using the potassa diluted as a local application to the membrane. I found that the potassa would clean the throat of the membrane ; but the soda would not check its formation. I then gave the potassa internally, to adults in doses of twenty drops, largely diluted, every three hours, until the disease begins to decline, which it has never failed to do within twenty-four hours ; then I diminish the dose, and I have never seen any bad effects from it.

I have discontinued using it as a local application. The membrane ceases to increase as soon as the system becomes affected by the medicine, and will, in a few days become detached. I am so well pleased with the action of this medicine, that I think it will prove to be a specific. I hope those having the disease to treat, will give it a fair trial.

---

